# Efficacy and Safety of Bariatric Surgery in Acquired Hypothalamic Obesity: A Systematic Review and Individual Patient Data Meta‐Analysis

**DOI:** 10.1111/obr.70182

**Published:** 2026-06-22

**Authors:** Johannes Fessler, Pauline Faucher, Christine Poitou, Carsten Dirksen

**Affiliations:** ^1^ Department of Endocrinology Copenhagen University Hospital – Amager and Hvidovre Copenhagen Denmark; ^2^ Nutrition Department, Reference Center for Rare Diseases PRADORT, Assistance Publique Hôpitaux de Paris Pitié‐Salpêtrière Hospital Paris France; ^3^ Inserm, Nutrition and Obesities: Systemic Approaches, NutriOmique Research Group Sorbonne Université Paris France; ^4^ Department of Clinical Medicine, Faculty of Health and Medical Sciences University of Copenhagen Copenhagen Denmark

**Keywords:** bariatric surgery, hypothalamic obesity, meta‐analysis

## Abstract

**Objective:**

This work aimed to investigate the efficacy and safety of Roux‐en‐Y gastric bypass (RYGB) and sleeve gastrectomy (SG) in adults with acquired hypothalamic obesity (HO).

**Design:**

Systematic review and meta‐analysis.

**Methods:**

Publications reporting relevant outcomes in patients with HO before and after RYGB or SG were identified through a systematic literature search. Two meta‐analyses were performed: one based on individual patient data (IPD) and one on case–control studies.

**Results:**

Thirteen publications including 36 unique patients with HO were included in the IPD meta‐analysis. Percent total weight loss was 16.5% (95% CI, 11.6%–21.4%; *p* = 5.7 × 10^−8^) after a median follow‐up of 31.5 months (IQR 24.0, 60.0). Weight loss after RYGB was slightly but not significantly larger compared with SG (17.4% vs. 15.2%). Type and rate of adverse events were reported to be as expected, and no major concerns were raised regarding hormone replacement therapy (HRT). Four publications including 49 patients with HO and 223 patients with common obesity were included in the case–control meta‐analysis that showed a 12.5%‐point lower weight loss in cases compared with controls with no major differences in the safety profile of the surgical procedures.

**Conclusions:**

Bariatric surgery in patients with acquired HO induces a clinically meaningful weight loss sustained for at least 2.5 years and appears to be safe. No significant differences were observed between RYGB and SG, but weight loss is substantially lower in patients with HO compared with patients with common obesity. However, the limited evidence calls for close surveillance if bariatric surgery is more widely adopted in this group of patients.

## 1 Introduction

Hypothalamic obesity (HO) is a rare condition caused by damage to the hypothalamus. It is characterized by hyperphagia, resulting in severe weight gain alongside an increased risk of endocrine, metabolic, cognitive, and behavioral disorders. Consequently, HO is associated with excess morbidity and mortality and decreased quality of life. The etiology of HO can be split into congenital abnormalities and acquired causes. The latter usually results from iatrogenic damage to the hypothalamic area following surgery or radiation therapy for cerebral tumors, most commonly craniopharyngiomas [1, 2].

The pathophysiology behind HO includes compromised function of satiety hormones in hypothalamic nuclei, leading to disturbed homeostatic and hedonic appetite regulation. Furthermore, hypothalamic damage leads to altered activity in the autonomic nervous system, with decreased sympathetic tone and increased parasympathetic tone resulting in lowered energy expenditure and hyperinsulinemia [2]. Related pituitary insufficiencies, for example, of the adrenal and thyroid axes, may also be associated with weight gain. In addition to these neuroendocrine changes, patients with hypothalamic damage may suffer from impaired impulse control, evoking episodic overeating, and lowered activity levels due to loss of initiative and sleep disturbances [1]. Thus, many mechanisms contribute to HO, making weight loss management complex and prone to fail even with newer gut hormone–based medications [3].

Bariatric surgery is the most effective weight loss intervention available and must also be considered for patients with acquired HO, but so far evidence is limited. In a review from 2013 including 21 patients with acquired HO, bariatric surgery resulted in an average weight loss of 20.9 and 15.1 kg after 6 and 12 months, respectively [4]. Later, a relatively large nationwide case–control study from France including 20 patients with HO found that Roux‐en‐Y gastric bypass (RYGB) or sleeve gastrectomy (SG), that is, the most widely used bariatric procedures, induced a percent total weight loss (%TWL) of 23% and 18% at 1‐ and 5‐year follow‐up, but this weight loss was significantly lower than in individually matched patients with common obesity (CO) (31% and 26%, respectively) [5]. This and three other case–control studies [6, 7, 8] were included in a recent meta‐analysis (52 cases and 256 controls) that showed a similar trend of lower weight loss in patients with HO compared with CO [9]. Yet, little is known about the relative efficacy and safety of RYGB and SG, which theoretically could differ due to the differential hormonal responses triggered by these two procedures [10]. Additionally, bioavailability of orally administered drugs may be affected by the altered gastrointestinal anatomy after bariatric surgery [11], including HRT, which is essential for patients with HO.

We, therefore, conducted this systematic review and meta‐analysis to investigate the efficacy and safety of bariatric surgery in adults with acquired HO including an individual patient data (IPD) meta‐analysis. Compared with previous analyses, our meta‐analysis offers an updated literature search, an extended follow‐up period, and allows for a direct comparison of surgical procedures after individual adjustment for preoperative age and BMI as well as follow‐up time.

## 2 Materials and Methods

The PRISMA guidelines for systematic reviews and meta‐analyses were followed [12].

### 2.1 Eligibility Criteria

For the IPD meta‐analysis, studies were eligible based on the following inclusion criteria: (1) population: adults (≥ 18 years of age at the time of bariatric surgery) with HO due to acquired hypothalamic damage; (2) intervention: bariatric surgery (restricted to RYGB or SG procedures); (3) outcomes: postoperative weight change with follow‐up of 12 months or longer. Exclusion criteria were as follows: (1) no original data reported; (2) insufficient data (i.e., missing data on age at time of bariatric surgery, type of surgery, postoperative weight change, or follow‐up time); and (3) IPD not available.

For the case–control meta‐analysis, eligibility was based on the following inclusion criteria: (1) population: patients with HO (cases) due to acquired hypothalamic damage and patients with CO (controls); (2) intervention: bariatric surgery (including all types of bariatric procedures); (3) outcomes: postoperative weight change with follow‐up of 12 months or longer. Exclusion criteria were as follows: (1) no original data reported; (2) number of cases < 5; (3) insufficient data (i.e., missing values on weight loss and associated standard deviations/confidence intervals or follow‐up time).

### 2.2 Search Strategy and Study Selection Process

**FIGURE 1:**
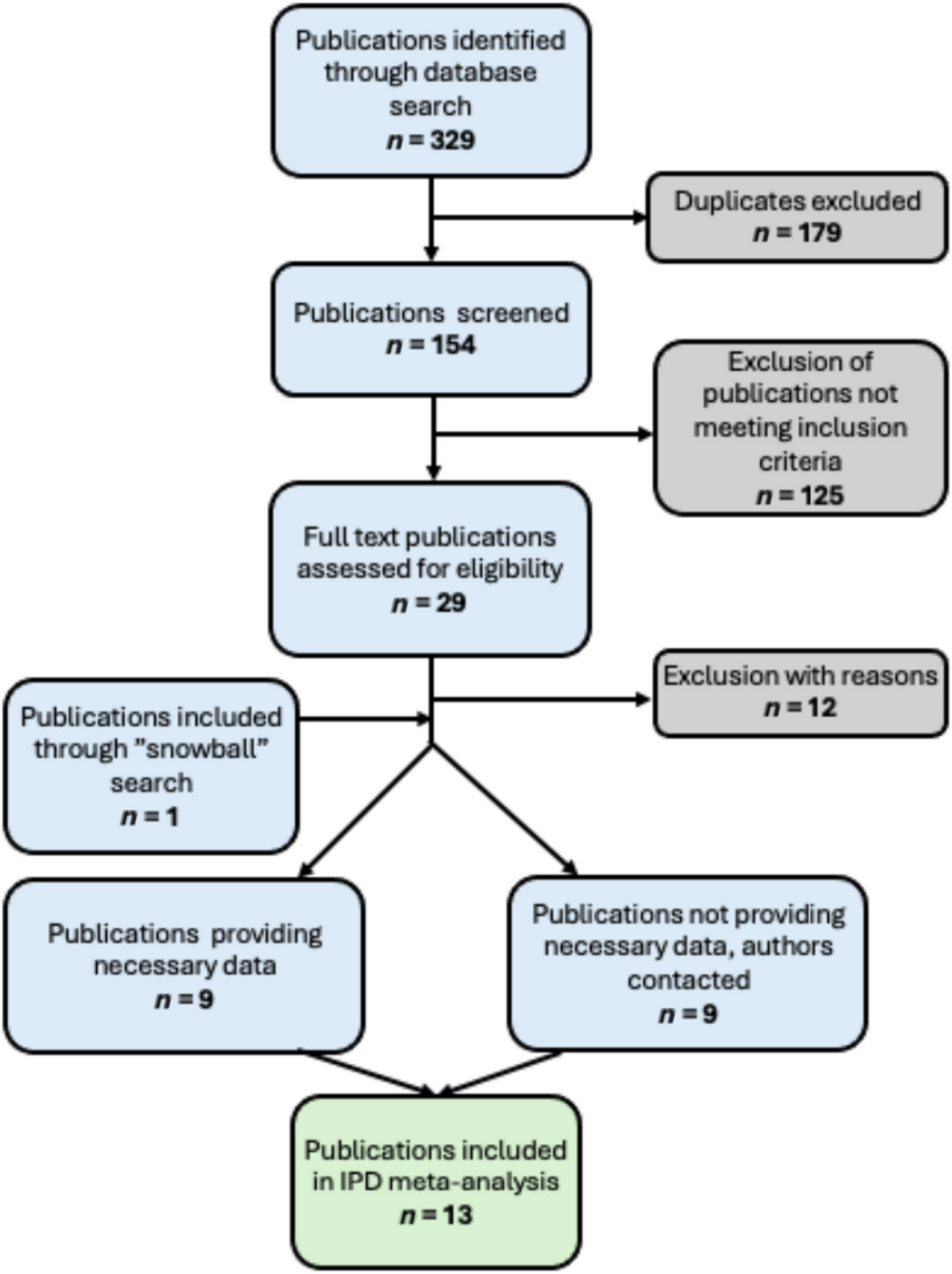
Study selection process flowchart for the individual patient data meta‐analysis.

**FIGURE 2:**
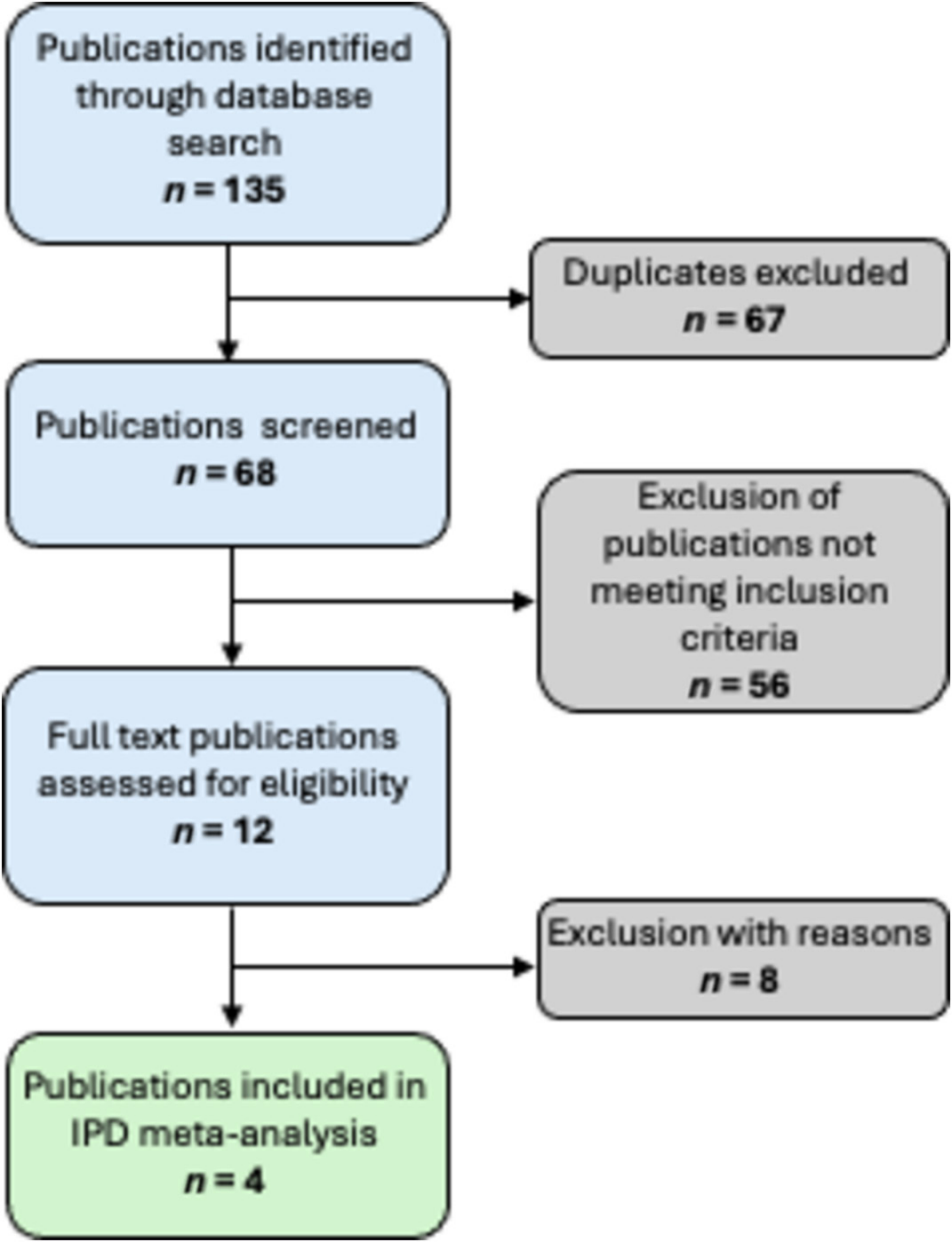
Study selection process flowchart for the case–control meta‐analysis.

Publications of interest were identified through a systematic literature search in the PubMed, Medline, Embase, and Web of Science databases carried out on May 9, 2026 (all search strings are available in Supporting Information S1). Search results were uploaded to Covidence systematic review software (Veritas Health Innovation, Melbourne, Australia) available at www.covidence.org. Duplicates were automatically removed before titles and abstracts of the identified publications were screened independently by two reviewers (J.I.F. and C.D.) according to the inclusion criteria. Publications considered relevant by both reviewers (in case of disagreement after a consensus decision) were subjected to full text review (*n* = 29). Using the snowball search method for publications selected for full‐text review, one additional publication that had not been identified in the literature search was included [13]. For the IPD meta‐analysis, full‐text review resulted in inclusion of 18 publications reporting original data on the efficacy and safety of bariatric surgery in patients with acquired HO. For the case–control meta‐analysis, the selection process resulted in four publications. A flowchart of the study selection processes is shown in Figures 1 and 2.

### 2.3 Data Collection and Outcomes

For the IPD meta‐analysis, the following variables were collected from the publications by one reviewer (JIF): year of publication, the study's country of origin, study design, patient characteristics (age at the time of bariatric surgery and etiology of hypothalamic damage), type of bariatric surgery, follow‐up time, and postoperative weight change (including if available preoperative and postoperative BMI and/or body weight). Of the included studies, nine publications either reported insufficient data (concerning a total of seven patients) or failed to report data on an individual patient level (57 patients). Except for one publication that explicitly stated that individual patient data would not be shared to protect the anonymity and privacy of patients [8], for publications with insufficient data, a minimum of two authors were contacted with an inquiry for providing the required data. For four publications (16 patients), the authors provided the requested data, and for five publications (28 patients), the authors did not respond to our request. For the latter, no data were included in the IPD meta‐analysis.

For the case–control meta‐analysis, the following variables were retrieved: number of cases and controls, mean %TWL, and standard deviations or confidence intervals. For one study [5], not all of the aforementioned variables were stated in numerical form and could not be obtained due to missing consent from cases. Therefore, the data were extracted from graphs using the PlotDigitizer software available at http://www.plotdigitizer.com. This method has been verified in a study investigating the validity and reliability of the software [14]. Also, we verified the method by comparing results obtained through the online tool to calculations on raw data on controls for the concerned study (discrepancy between numerical value of variables obtained through the two methods was on average 0.3%).

### 2.4 Risk of Bias Assessment

Ten out of 13 studies included in the IPD meta‐analysis were case reports or series, which introduces a high risk of bias.

### 2.5 Statistical Analysis

For the IPD meta‐analysis, the primary outcome of interest was total weight loss percentage (%TWL) and was calculated as percentage change in body weight (kg) or BMI (kg/m^2^) from before to last follow‐up after bariatric surgery. For studies reporting body weight (all except one), we also report total weight loss (TWL) calculated by subtracting the body weight at last follow‐up from the preoperative value. Excess weight loss percentage (%EWL) was calculated using the formula (BMI_pre_ − BMI_post_)/(BMI_pre_ − 25 kg/m^2^) × 100% and used to identify patients with a suboptimal clinical response (%EWL ≤ 50) [15]. Outcomes (%TWL and TWL) were found to comply with the assumption of normal distribution by visual inspection (histograms/Q–Q plots) and were consequently analyzed using one‐sample Student's *t*‐test. To compare postoperative weight loss between RYGB and SG, we used a linear regression model adjusted for age at time of bariatric surgery, preoperative BMI, and follow‐up time (results of linear model are available in Supporting Information S2).

For the case–control meta‐analysis, standard deviations were calculated if only confidence intervals were reported [16].

Median and IQR are reported if not otherwise specified. A *p* value < 0.05 was set to be significant. Statistical analyses were performed in R Version 4.2.3 (http://www.Rproject.org) using the “meta” package.

## 3 Results

### 3.1 IPD Meta‐Analysis

Thirteen publications published between 2007 and 2023, of which most were case reports (*n* = 5) or case series (*n* = 5), were included in the IPD meta‐analysis (see Table 1). Most publications were from Europe with only two case reports from the United States and one from Japan. Data on a total of 36 unique patients were extracted; median age was 29 years, and BMI was 46.7 kg/m^2^. More patients had undergone RYGB (*n* = 22) than SG (*n* = 14), and median postoperative follow‐up exceeded 2.5 years (see Table 2). Postoperative weight loss (%TWL) was 16.5%, equivalent to a TWL of 25.6 kg (see Table 2). A suboptimal clinical response (defined as %EWL ≤ 50%) was observed in more than two‐thirds of patients. Despite numeric differences, %TWL did not differ between the two surgical procedures (2.9% [95% CI, −7.7% to 13.6%, *p* = 0.6] lower after SG compared with RYGB).

**TABLE 1:** Characteristics of publications included in the individual patient data meta‐analysis of the efficacy of bariatric surgery in patients with acquired hypothalamic obesity.

First author, year of publication, country of origin	Study design, follow‐up period	Patient characteristics, surgery type	%TWL (median)
Dischinger et al. [17], 2023, Germany	Case–control study (prospective) Median follow‐up of 47 months	7 cases with CP (1 of which possibly astrocytoma, not CP) RYGB = 4. SG = 3	3.9%
Schultes et al. [18], 2023, Switzerland	Case series Median follow‐up of 96 months	3 cases with CP RYGB = 3	26.2%
Faucher et al. [5], 2022, France	Case–control study (retrospective) Median follow‐up of 60 months	6 cases with CP RYGB = 3. SG = 3	17.3%
Garrez et al. [6], 2020, Belgium	Case–control study (retrospective) Median follow‐up of 28 months	2 cases with CP RYGB = 2	32.6%
Nezu et al. [19], 2019, Japan	Case report Follow‐up 15 months	1 case with IG SG = 1	27.9%
Trotta et al. [20], 2017, Italy	Case series Follow‐up of 24 months	3 cases with CP SG = 3	25.0%
Bretault et al. [21], 2016, France	Case series Follow‐up of 18 months	3 cases with CP RYGB = 2. SG = 1	9.0%
Wolf et al. [22], 2016, Austria	Case series Median follow‐up of 17 months	3 cases with CP RYGB = 3	32.3%
Gatta et al. [23], 2013, France	Case series Median follow‐up of 39 months	3 cases with CP, 1 case with choristoma RYGB = 2. SG = 2	11.9%
Page‐Wilson et al. [24], 2013, USA	Case report Follow‐up of 15 months	1 case with CP RYGB = 1	24.2%
Weismann et al. [25], 2013, Germany	Case report Follow‐up of 24 months	1 case with CP SG = 1	−3.4%
Pappis et al. [26], 2012, Greece	Case report Follow‐up of 24 months	1 case with CP RYGB = 1	37.5%
Inge et al. [27], 2007, USAss	Case report Follow‐up of 30 months	1 case with CP RYGB = 1	21.9%

Abbreviations: %TWL, percent total weight loss; CP, craniopharyngioma; IG, intracranial germinoma.

**TABLE 2:** Results from the individual patient data meta‐analysis of the efficacy of bariatric surgery in patients with acquired hypothalamic obesity.

	All	RYGB	SG
Patients	36 from 13 studies	22 from 10 studies	14 from 7 studies
Preoperative BMI [median (IQR)]	46.7 (43.1, 54.3)	46.7 (42.4, 52.6)	45.9 (43.3, 56.4)
Age at BS [median (IQR)]	29.0 (21.75, 41.5)	29.5 (23.5, 44.0)	26.5 (21.3, 37.5)
Follow‐up [median (IQR)]	31.5 (24.0, 60.0)	37.5 (25.0, 60.0)	30.0 (24.0, 60.0)
TWL kg [mean (95% CI)]	25.64 (18.62–32.67)***	28.11 (19.48–36.74)***	21.53 (8.00–35.05)**
%TWL [mean (95% CI)]	16.52 (11.64–21.41)***	17.40 (10.40–24.39)***	15.15 (7.93–22.36)***
Suboptimal clinical response	25 (69.4%)	15 (68.2%)	10 (71.4%)

Abbreviations: %TWL, percent total weight loss; BMI, body mass index (kg/m^2^); BS, bariatric surgery; CI, confidence interval; RYGB, Roux‐en‐Y gastric bypass; SG, sleeve gastrectomy.

* *p* < 0.05.

** *p* < 0.01.

*** *p* < 0.001.

Type and frequency of adverse events and complications were, overall, reported to be as expected following bariatric surgery [5]. Specific changes in HRT were only reported in four publications [19, 20, 22, 26] (*n* = 8). Postoperative hydrocortisone dose was reported to be unchanged (*n* = 5) or slightly increased (*n* = 3), and no cases of adrenal crisis were reported. Of six patients, levothyroxine dose was unchanged in three, decreased in two, and increased in one case.

### 3.2 Case–Control Meta‐Analysis

**FIGURE 3:**
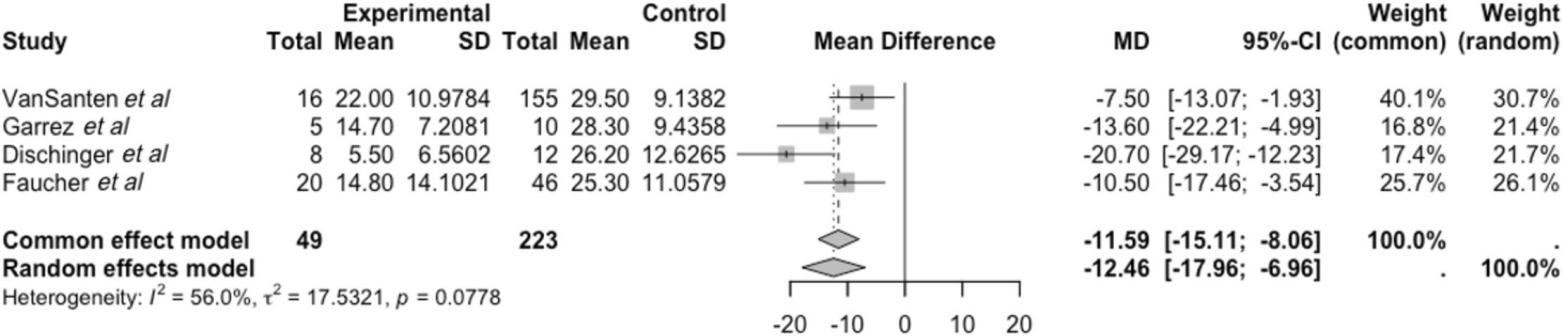
Percent total weight loss in matched patients with hypothalamic obesity (experimental) or common obesity (control) following bariatric surgery.

Four publications including matched patients with HO (*n* = 49 cases) and CO (*n* = 223 controls) were included. The ratio between cases and controls varied notably across the studies (from 1:1.5 to 1:9.7). Follow‐up was 24–62 months. Using a random effects model, %TWL following bariatric surgery was 12.5% lower in patients with HO compared with those with CO (see Figure 3). Two case–control studies reported a similar rate of serious adverse events among cases with HO and controls with CO [5, 6].

## 4 Discussion

Bariatric surgery may lead to clinically meaningful weight loss in patients with HO, but evidence mostly relies on case reports or case series [4, 5, 6, 7, 8, 17, 18, 19, 20, 21, 22, 23, 24, 25, 26, 27, 28, 29]. In this systematic review, we provide the largest set of individual patient data to date (*n* = 36) and find a mean weight loss of 16.5% after a median follow‐up period of more than 2.5 years with no difference in weight loss between the RYGB and SG procedures. However, only 11 of 36 patients experienced a good clinical weight loss response when defined as %EWL greater than 50%, and in the meta‐analysis of case–control studies, we confirm that the weight loss after bariatric surgery in patients with HO is markedly lower (12.5 percentage points) than in matched patients with CO.

Our analysis, thereby, extends previous reviews within the field. An earlier systematic review and IPD meta‐analysis from 2013 identified 21 patients with HO due to previous treatment for craniopharyngioma that had undergone bariatric surgery and had follow‐up after 6 and/or 12 months [4]. At 12 months, weight loss was 6.1%, 19.6%, 20.2%, and 24.8% following gastric banding (*n* = 5), SG (*n* = 6), RYGB (*n* = 6), and biliopancreatic diversion (*n* = 1), respectively. The slightly higher weight loss following RYGB and SG in this analysis compared with ours could be due to differences in the length of the follow‐up period (≤ 12 months vs. ≥ 12 months) [29, 30]. More recently, a systematic review and meta‐analysis of case–control studies with an identical design as ours found weight loss to be 6.2%, 7.7%, and 7.7% lower in patients with HO compared with matched patients with CO at 12, 24, and 60 months follow‐up after bariatric surgery. The somewhat larger difference between cases and controls in our meta‐analysis may be explained by variations in study selection and data extraction. Thus, in the previously published meta‐analysis, the authors included two publications [7, 8] from the same research group with eight duplicated cases (nearly 20% of all cases in their analysis) and apparently did not account for this in their analysis. In our meta‐analysis, we excluded these duplicates and included data from one additional case–control study [17].

The lower postoperative weight loss in patients with HO is also reflected in the relatively high percentage of patients not reaching a clinically optimal response, as defined by a %EWL of 50% or higher. Hence, in our IPD meta‐analysis, 69% of patients with HO failed to reach this goal, which is much higher than what would typically be expected in patients with CO; for example, a study of 658 individuals with CO only found 24% to have a suboptimal weight loss response after a median follow‐up of ~4.5 years following RYGB or SG [15]. Notably, long‐term suboptimal weight loss responses might indeed be even higher in patients with HO, because follow‐up was < 2 years in seven of 36 cases in our IPD meta‐analysis, and it is well known that weight loss after bariatric surgery typically reaches its maximum around 1–2 years postoperatively, often followed by some weight regain [29, 30].

The relatively large number of patients undergoing either RYGB (61%) or SG (39%) in our IPD meta‐analysis permitted a comparison of weight loss between the two procedures, which appears to be largely comparable although this result is associated with a relatively large uncertainty as indicated by a wide confidence interval. However, if true, this finding is in line with the general notion based on randomized controlled trials in patients with CO that RYGB produces a slightly, but often not clinically importantly, larger weight loss than SG [30, 31]. Importantly, our dataset did not allow for assessment of procedure‐specific complications such as the incidence of internal herniation, postoperative gastroesophageal reflux disease, or the need for revisional surgery, which would typically also influence clinical decisions.

Several factors can be speculated to lead to lower efficacy of bariatric surgery in patients with HO. As the mechanism of weight loss after RYGB and SG in CO relies on changes in neurohormonal signaling, it seems plausible that the lower efficacy in HO may be explained by either impaired afferent signaling to the hypothalamus or impaired response to the hormonal changes. One case–control study including eight patients with HO found that postprandial circulating levels of the satiety hormones peptide‐YY and glucagon‐like peptide‐1 (GLP‐1) are also higher in patients with HO who have undergone bariatric surgery compared with unoperated patients with HO or CO [17] (increased postprandial satiety gut hormones is a well‐known hallmark of bariatric surgery) [32]. Surprisingly, in patients with HO, postprandial GLP‐1 levels at 60 min were negatively correlated with satiety, which could be interpreted as a disturbed hormonal appetite regulation despite normal secretion. Another observation from our IPD analysis is the wide range in %TWL (−30% to 41%), which seems to be an unusually large variation in the response to bariatric surgery that could be related to heterogeneity in the extent and degree of hypothalamic damage causing varying sensitivity to the postoperative changes in satiety hormones. A better understanding on the implications of specific hypothalamic lesions may in future help identify patients who will benefit best from specific procedures or interventions. Interestingly, it has been suggested that responses to treatment with GLP‐1 receptor agonist may predict the outcome of bariatric surgery in patients with HO; for example, if pharmaceutical treatment fails, then the hormonal changes elicited by surgery would be predicted to be less effective [17]. This hypothesis, however, warrants formal testing, and variations in hormone sensitivity may only be part of the explanation for the lower efficacy of bariatric surgery in patients with HO as various clinical domains may play a role: psychosocial disorders, hyperphagia, sleep disturbances, decreased energy expenditure, hyperinsulinemia, and hypopituitarism [2].

Postoperative changes in gastrointestinal anatomy may affect drug bioavailability [11], which is of particular relevance in patients with HO where most suffer from hypopituitarism and are dependent on HRT [2]. Especially replacement therapy with oral hydrocortisone for patients with adrenal insufficiency is vital. It is therefore reassuring that no cases of severe hydrocortisone deficiencies, that is, adrenal crisis, were reported and that the authors, in general, conclude that bariatric surgery seems to be safe with regard to HRT.

The emergence of new pharmacotherapeutics, particularly gut hormone receptor agonists (such as GLP‐1 and GIP analogs) and MC4R agonists, may offer a promising perspective for the management of HO. Accumulating data suggest that they may help address the central dysregulation of appetite and energy balance, which characterizes this condition, yet evidence among patients with HO is still scarce [33]. Thus, it is necessary to re‐evaluate therapeutic strategies and promote individualized, multimodal approaches [34]. In this context, bariatric surgery should be integrated into a comprehensive and long‐term management plan. Given the complex and chronic nature of HO, surgical intervention must be accompanied by continuous multidisciplinary care including nutritional support, behavioral and cognitive approaches, psychological and emotional follow‐up, promotion of physical activity, and attention to sleep and circadian rhythms. Such an integrative approach is essential to optimize and sustain clinical outcomes over time. Ultimately, the most effective management of HO may rely on a combination of therapeutic modalities [34], tailored to each patient's clinical profile and adapted over time to evolving needs.

### 4.1 Strengths and Limitations of the IDP Meta‐Analysis

Despite representing the largest individual patient dataset on weight loss after bariatric surgery in patients with HO to date, we acknowledge that our analysis has important limitations. Firstly, given that the dataset mainly relies on case reports, case series, and case–control studies, there is a high risk of reporting bias, which could lead to an overestimation of the treatment benefits and/or an underestimation of risk of complications and adverse events. Further, because data have been collected from many different surgical centers in several countries, the surgical procedures may not be standardized, and we do not have data on the experience of the surgeons. This, together with the relatively large range in postoperative follow‐up (15–96 months), may contribute to the substantial variability in the magnitude of weight loss observed. It is, therefore, important to emphasize that the level of evidence reported here should not be treated as equivalent to that obtained from a meta‐analysis based on randomized or even comparative observational studies. Secondly, the IPD analysis might be prone to selection bias, as it is very likely that patients described in the identified publications are not representative of the entire population of patients with acquired causes of HO. Thirdly, it should be kept in mind that we chose to focus only on patients with acquired causes of HO and mainly identified cases caused by craniopharyngiomas (34 of 36 cases). It is therefore unclear whether our findings can be applied to patients with other causes of HO or even just all patients with prior craniopharyngiomas because hypothalamic damage may show high interindividual variability. Fourthly, our cohort is too small, and follow‐up is too short to make any firm conclusions with respect to the long‐term safety of bariatric surgery in patients with HO.

## 5 Conclusion

This systematic review and meta‐analyses indicate that bariatric surgery in adults with acquired HO induces a clinically meaningful weight loss that is maintained at more than 2.5 years of follow‐up, although the weight loss appears to be lower than in matched patients with CO. No statistically significant differences were seen between RYGB and SG, and both procedures seem to be safe without major impact on dosage or efficacy of HRT. Thus, based on the currently available evidence, bariatric surgery should be considered also for patients suffering from HO. However, the lack of larger well‐performed clinical trials is a major caveat, and the low prevalence of HO makes it difficult to conduct such trials; therefore, systematic monitoring of outcomes following bariatric surgery in patients with HO should be prioritized both by national and international authorities.

## Author Contributions

J.F. and C.D. performed conception and design of research. J.F. collected data. J.F. and C.D. screened papers. P.F. and C.P. provided additional data. J.F. analyzed data. J.F., P.F., C.P., and C.D. interpreted results. J.F. drafted manuscript. J.F., P.F., C.P., and C.D. edited and revised manuscript, and approved final version of the manuscript.

## Conflicts of Interest

J.F., P.F., and C.P. declare no conflicts of interest. C.D. has, on behalf of his institution, received research funding from the Novo Nordisk Foundation and over the past 3 years received personal fees (lecturer, consulting, committee member) from Novo Nordisk, AstraZeneca, Amylyx, and Empros and served in various unpaid roles (lecturer, chairperson, principal investigator) for Novo Nordisk, Amgen, AstraZeneca, and Eli Lilly. None of these commitments has had any influence on the present work.

## Supporting information


**Data S1:** Supporting Information.

## Data Availability

The data that support the findings of this study are available on request from the corresponding author. The data are not publicly available due to privacy or ethical restrictions.
