# Supplementary material for: Efficacy and Safety of Bariatric Surgery in Acquired Hypothalamic Obesity: A Systematic Review and Individual Patient Data Meta‐Analysis
Source: Obes Rev. 2026 Jun 22;27(9):e70182. doi: 10.1111/obr.70182 (PMC13460666; doi:10.1111/obr.70182)
Supplement: Supplementary file 1 [file OBR-27-e70182-s001.pdf]

Supplementary material

Efficacy and safety of bariatric surgery in acquired hypothalamic obesity – a systematic review and individual patient data meta-analysis

J. Fessler (johannes607@hotmail.com). P. Faucher. C. Poitou. C. Dirksen.

S1: Search strings for the individual-patient-data meta-analysis

PubMed:
("Bariatric Surgery"[MeSH Terms] OR "bariatric surger*"[All Fields] OR "gastric sleeve*"[All Fields] OR "sleeve gastric*"[All Fields] OR "gastric bypass*"[All Fields] OR "roux-en-y"[All Fields] OR "RYGB"[All Fields]) AND ("hypothalamic syndrome"[All Fields] OR "hypothalamic obes*"[All Fields])

Web of Science:
ALL=(("Bariatric Surgery" OR "bariatric surger*" OR "gastric sleeve*" OR "sleeve gastric*" OR "gastric bypass*" OR "roux-en-y" OR RYGB) AND ("hypothalamic syndrome" OR "hypothalamic obes*"))

Medline and Embase:
(exp Bariatric Surgery OR bariatric surger* OR gastric sleeve* OR sleeve gastric* OR gastric bypass* OR roux-en-y OR RYGB) AND (hypothalamic syndrome OR hypothalamic obes*).mp

S1: Search strings for the case-control meta-analysis

PubMed:
("Bariatric Surgery"[MeSH Terms] OR "bariatric surger*"[All Fields]) AND ("hypothalamic syndrome"[All Fields] OR "hypothalamic obes*"[All Fields]) AND ("case-control"[All Fields] OR "control*"[All Fields])

Web of Science:
ALL=(("bariatric surger*") AND (“hypothalamic syndrome” OR “hypothalamic obes*”) AND ("case-control" OR "control*"))

Medline and Embase:
(exp Bariatric Surgery OR bariatric surger*) AND (hypothalamic syndrome OR hypothalamic obes*) AND (“case-control” OR “control*”).mp

S2: "R" code and output from linear model
Response variable: %TWL.
Predictor variables: Type of surgery, BMI prior to surgery, age at the time of surgery, length of follow-up.

```
lm(BMIWeightloss_prc~Type+Age_BS+Follow.up_months+BMI_pre)
```

```
Coefficients:
                  Estimate Std. Error t value Pr(>|t|)
(Intercept)       0.2855177  0.1761008   1.621    0.115
TypeSG           -0.0293129  0.0521521  -0.562    0.578
Age_BS           -0.0009794  0.0022995  -0.426    0.673
Follow.up_months -0.0005377  0.0008505  -0.632    0.532
BMI_pre          -0.0010790  0.0028354  -0.381    0.706

Residual standard error: 0.1508 on 31 degrees of freedom
Multiple R-squared:  0.03416,   Adjusted R-squared:  -0.09047
F-statistic: 0.2741 on 4 and 31 DF,  p-value: 0.8924
```

```
                        2.5 %      97.5 %
(Intercept)      -0.073642252 0.644677608
TypeSG           -0.135677676 0.077051942
Age_BS           -0.005669205 0.003710344
Follow.up_months -0.002272265 0.001196948
BMI_pre          -0.006861836 0.004703822
```
